# Cerebral oxygen saturation and cerebrovascular instability in newborn infants with congenital heart disease compared to healthy controls

**DOI:** 10.1371/journal.pone.0251255

**Published:** 2021-05-10

**Authors:** Nhu N. Tran, Jodie K. Votava-Smith, John C. Wood, Ashok Panigrahy, Choo Phei Wee, Matthew Borzage, S. Ram Kumar, Paula M. Murray, Mary-Lynn Brecht, Lisa Paquette, Kenneth M. Brady, Bradley S. Peterson

**Affiliations:** 1 Institute for the Developing Mind, The Saban Research Institute, Children’s Hospital Los Angeles, Los Angeles, California, United States of America; 2 Department of Surgery, Keck School of Medicine, University of Southern California, Los Angeles, California, United States of America; 3 Division of Cardiology, Children’s Hospital Los Angeles, Los Angeles, California, United States of America; 4 Keck School of Medicine, University of Southern California, Los Angeles, California, United States of America; 5 University of Pittsburgh Medical Center, Children’s Hospital of Pittsburgh, Pittsburgh, Pennsylvania, United States of America; 6 Department of Pediatric Radiology, Children’s Hospital Los Angeles, Los Angeles, California, United States of America; 7 Department of Preventive Medicine, Southern California Clinical and Translational Science Institute, Keck School of Medicine, University of Southern California, Los Angeles, California, United States of America; 8 Division of Neonatology, Department of Pediatrics, Fetal and Neonatal Institute, Children’s Hospital Los Angeles, Los Angeles, California, United States of America; 9 Division of Cardiothoracic Surgery, Children’s Hospital Los Angeles, Los Angeles, California, United States of America; 10 Institute for Nursing and Interprofessional Research, Children’s Hospital Los Angeles, Los Angeles, California, United States of America; 11 School of Nursing, University of California, Los Angeles, Los Angeles, California, United States of America; 12 Lurie Children’s Hospital of Chicago, Anesthesiology and Pediatrics, Northwestern University Feinberg School of Medicine, Chicago, Illinois, United States of America; 13 Department of Psychiatry, Keck School of Medicine, University of Southern California, Los Angeles, California, United States of America; Kobe University Graduate School of Medicine School of Medicine, JAPAN

## Abstract

**Objective:**

Infants with Congenital Heart Disease (CHD) are at risk for developmental delays, though the mechanisms of brain injury that impair development are unknown. Potential causes could include cerebral hypoxia and cerebrovascular instability. We hypothesized that we would detect significantly reduced cerebral oxygen saturation and greater cerebrovascular instability in CHD infants compared to the healthy controls.

**Methods:**

We performed a secondary analysis on a sample of 43 term infants (28 CHD, 15 healthy controls) that assessed prospectively in temporal cross-section before or at 12 days of age. CHD infants were assessed prior to open-heart surgery. Cerebral oxygen saturation levels were estimated using Near-Infrared Spectroscopy, and cerebrovascular stability was assessed with the response of cerebral oxygen saturation after a postural change (supine to sitting).

**Results:**

Cerebral oxygen saturation was 9 points lower in CHD than control infants in both postures (β = -9.3; 95%CI = -17.68, -1.00; p = 0.028), even after controlling for differences in peripheral oxygen saturation. Cerebrovascular stability was significantly impaired in CHD compared to healthy infants (β = -2.4; 95%CI = -4.12, -.61; p = 0.008), and in CHD infants with single ventricle compared with biventricular defects (β = -1.5; 95%CI = -2.95, -0.05; p = 0.04).

**Conclusion:**

CHD infants had cerebral hypoxia and decreased cerebral oxygen saturation values following a postural change, suggesting cerebrovascular instability. Future longitudinal studies should assess the associations of cerebral hypoxia and cerebrovascular instability with long-term neurodevelopmental outcomes in CHD infants.

## Introduction

Infants with Congenital Heart Disease (CHD) are at high risk for developmental delays [1–3], with up to 75% experiencing delays in cognitive or motor development in pre-school to school-age years [4, 5], and 65% requiring remedial academic or behavioral services [6]. Although advances in medical and surgical techniques have improved survival from CHD, those innovations have not produced dramatic improvements in neurodevelopmental outcomes. Moreover, large multicenter studies have excluded surgical factors as independent predictors of delays [1, 7]. Therefore, other potential causes of developmental delays must be identified.

Two potential causes of delayed development that have not been examined extensively include cerebral hypoxia [8] and cerebrovascular instability [9, 10]—i.e., dysfunction of the brain’s homeostatic response that maintains stable cerebral blood flow during a change in posture. Cerebrovascular instability causes fluctuating blood oxygen levels in the brain and may reduce cerebral blood flow and nutrient delivery, thereby placing the brain at risk for functional impairment and structural damage. Cerebral hypoxia and impaired perfusion from cerebrovascular instability are plausible consequences of the profoundly altered blood flow and systemic hypoxemia associated with CHD. Thus, we assessed these characteristics in CHD and healthy control infants, hypothesizing the detection of significantly reduced cerebral oxygen saturation and greater cerebrovascular instability in CHD compared to healthy control infants.

## Materials and methods

We performed a secondary analysis on a cross-sectional study comparing CHD and healthy infants [11], using more sophisticated statistical modeling and more informative analyses than the primary analysis, and included covariates that it did not consider. We obtained written informed consent from all parents of infants included in this study. All procedures contributing to this work comply with the ethical standards of the relevant national guidelines on human experimentation (Good Clinical Practice) and with the Helsinki Declaration of 1975, as revised in 2008, and has been approved by the institutional committees (Committee on Clinical Investigations of Children’s Hospital Los Angeles and AltaMed ethics committee). Recruitment and assessments were performed between March 2015 and December 2016.

### Sample ascertainment

#### CHD infants

We recruited eligible pregnant women carrying fetuses diagnosed with CHD and who were patients of our Fetal Maternal Center and Fetal Cardiology clinics. We also recruited eligible infants with CHD consecutively admitted to our cardiothoracic intensive care and inpatient units. Inclusion criteria for infants with CHD included: (1) postnatal age ≤12 days and did not yet have open-heart surgery; (2) ≥37 weeks gestational age at birth; and (3) a documented structural heart defect requiring neonatal admission to Children’s Hospital Los Angeles for monitoring or intervention. Exclusion criteria included: (1) hemodynamic instability (dopamine greater than or equal to 5 mcg/kg/min) or (2) endotracheal intubation.

#### Healthy infants

We recruited healthy pregnant women through recruitment flyers and healthy eligible infants from our newborn clinics. Inclusion criteria for healthy infants included: (1) postnatal age ≤12 days; (2) ≥37 weeks gestational age at birth; (3) no major prenatal, delivery, or postnatal complications; and (4) uncomplicated neonatal course.

#### Additional exclusion criteria for both groups

These included documented: (1) genetic defects except 22q11.2 deletion syndrome in the CHD infants; (2) congenital anomalies other than CHD; (3) receiving antibiotics for a known infection; (4) diagnosis of intrauterine growth restriction or small for gestational age; (5) intraventricular hemorrhage; (6) maternal substance abuse; (7) maternal chorioamnionitis; (8) neurologic abnormalities defined by cranial ultrasound or MRI; (9) last trimester maternal or neonatal use of steroids.

### Participating infants

We consented 29 healthy controls and 34 infants with CHD. Of these, 14 healthy infants were removed from the study because they did not return to the hospital within 12 days (n = 13) or due to data loss (n = 1); 6 CHD infants were withdrawn from the study because they were intubated (n = 5) or died (n = 1). Our final study sample comprised of 43 infants (15 controls, 28 CHD) at 37–44 weeks gestational age who had usable near-infrared spectroscopy (NIRS) data.

### Clinical data

A neonatal intensive care registered nurse extracted information from the obstetrical and neonatal records onto a standardized medical abstraction form. Information included pregnancy, labor, and delivery notes, laboratory measures, and neonatal course (e.g., Apgar scores and physical exam).

### Cerebral oxygen saturation measures

We measured cerebral oxygen saturation using an INVOS 5100C NIRS monitor (Somanetics, Troy, MI). Cerebral oxygen saturation measured with NIRS reflects tissue oxygenation in the capillary, venous, and arterial vasculature, which is weighted to the venous blood (roughly 70–80%) [12–14]. We also measured pulse oximetry, which requires pulsatile flow and therefore estimates arterial oxygenation [15]. First, we ensured the infant was in a comfortable resting state (e.g., provided a pacifier), then placed a neonatal cerebral oxygen saturation sensor on the center of the infant’s forehead and a preductal pulse oximetry sensor on the right hand per manufacturer guidelines. We connected the monitors to a Bernoulli data acquisition system (Cardiopulmonary Corporation, Milford, CT), which acquired heart rate, respiratory rate, and arterial blood pressure (for infants with invasive arterial lines) or cuff blood pressure from the Philips Intellivue MP70 monitors. Only heart rate, respiratory rate, and arterial blood pressure (for infants with invasive arterial lines) were sampled every 5 seconds by the Bernoulli data acquisition system. Infants with cuff blood pressures had 1 measurement before the postural change.

We followed standard operating procedures developed specifically for the postural change. We placed the bed of the radiant warmer in a horizontally flat position, swaddled the infant, and placed him/her in the supine (0⁰) posture to acquire measures continuously for 5 minutes. Infants were then placed in a standard 90° sitting posture, with one hand maintaining erectness of the back and spine, and the other hand preserving erectness of the head, neck, and chin. Data acquisition continued for another 5 minutes in the sitting posture. The standard sitting technique ensured that infants were not slouching nor hunched while in the sitting posture to minimize any potential abdominal pressure or compression. Infants were required to be calm during NIRS measurements to avoid distress-induced intrathoracic pressure changes that could affect blood pressure; in rare instances when they were not calm during the postural change, they were allowed to settle while sitting for 20–30 seconds during the NIRS recording.

### Cerebral oxygen saturation time series

**Fig 1:**
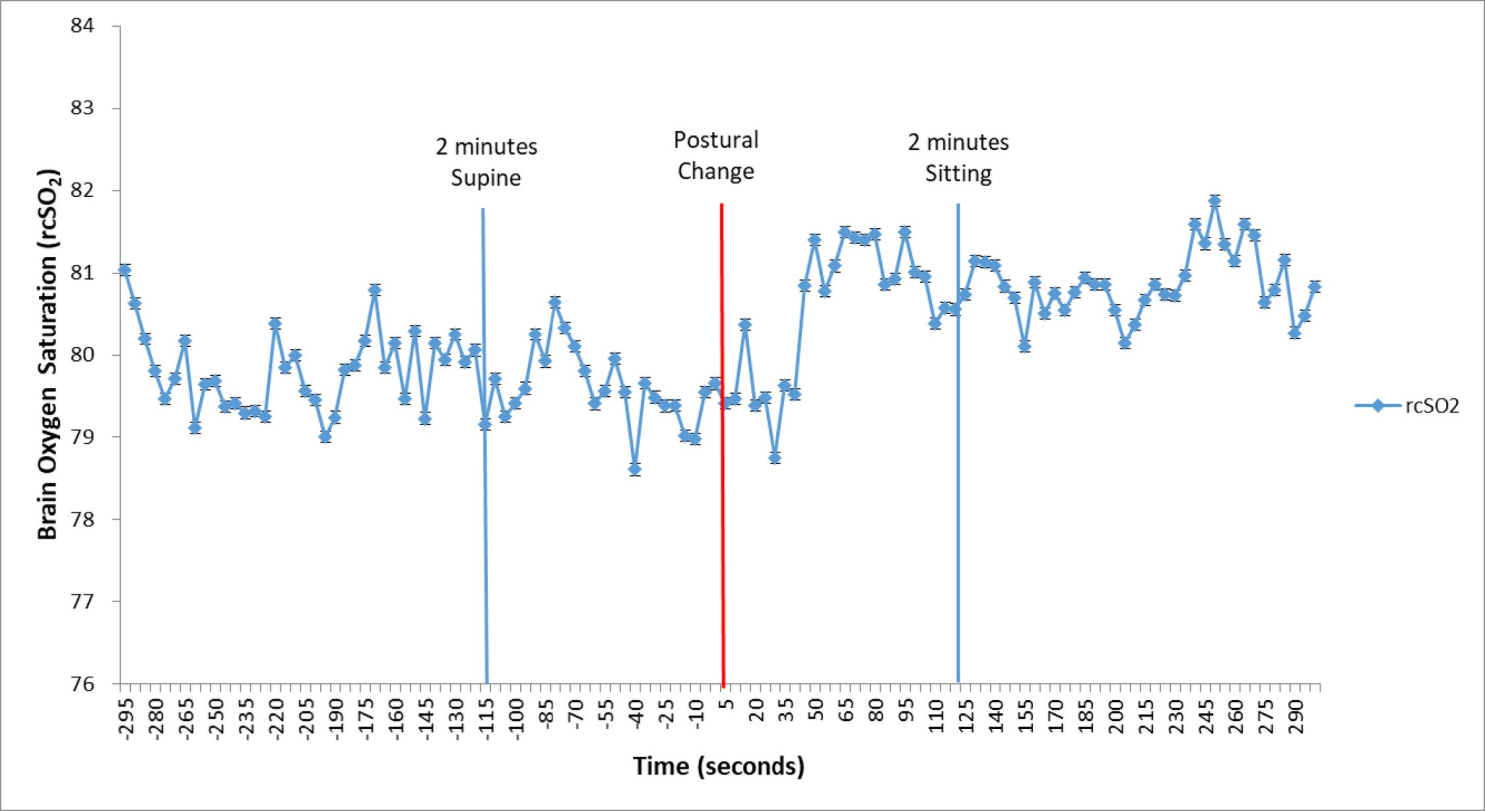
Example of a rcSO_2_ time series. We show the mean rcSO_2_ time series for the healthy control infants. Measures were collected every 5 seconds for 5 minutes in each posture. Visually examining all individual time series provided the basis for selecting values 2 minutes before and after the postural change for our cerebrovascular stability analysis. We aimed to capture posture-induced changes, but eliminate the eventual return to baseline after the change.

We visually assessed cerebral oxygen saturation values 5 minutes before and 5 minutes after the postural change to determine the timeframe that would accurately capture the baseline and post-postural cerebral oxygen saturation response to the change in posture. We concluded empirically that the last 2-minutes of the supine cerebral oxygen saturation values and the first 2-minutes of the sitting cerebral oxygen saturation values would be suitable to capture the cerebral oxygen saturation response to the postural change (**Fig 1**). In addition to this empirical determination of recording duration from the time series, a physiologic rationale supports use of the 2-minute duration. When cerebral autoregulation was modeled as a Butterworth high pass filter using piglet data, the cutoff frequency for the autoregulation response was determined to be 30 seconds. A full autoregulatory response was seen in that study at 60 seconds. Therefore, a 2-minute window provides adequate time for an autoregulatory response to occur [16]. Although we are not measuring cerebral autoregulation because we do not have correlations of NIRS with blood pressure, we used this timeframe to guide our cerebrovascular stability response.

### Statistical analyses

We used Stata 13.1 (Statacorp, College Station, TX) for all statistical analyses. A power analysis from G*Power 3.1 [17] indicated that a total sample size of 46 subjects (or 23 per group) would detect a large effect size (approximate d = 0.6) with an alpha of 0.05 and power of 0.80 using a repeated measures analysis of variance comparing cerebrovascular instability between groups. Large effect sizes have been detected in previous studies comparing cerebral blood flow and injury in CHD and control infants [18–20]. Based on the effect size from brain oxygenation levels between groups rcSO_2_% in **Table 1**, we could detect an effect of *r* = 0.54 or Cohen’s *d* = 1.30 in our repeated measures analysis.

**Table 1:** Infant demographics and physiologic measures.

	Healthy N = 16			CHD N = 28			Test Statistic	df	p*-*value
	Mean	± SD	N	Mean	± SD	N			
**Postnatal Age (days)**	6.9	2.6	16	2.9	2.8	28	4.6	42	0.001*
**Weight (kg)**	3.29	0.39	15	3.36	0.61	28	-0.4	41	0.70
**Birth Length (cm)**	50	2.7	15	50	0.1	28	-0.3	41	0.70
**Birth Head Circ. (cm)**	34.5	1.36	10	34.4	0.09	28	0.1	36	0.92
**Birth Gest. Age (weeks)**	39	1.16	15	39	0.94	28	0.2	41	0.87
**Postconceptional Age (weeks)**	40	1.21	15	39.4	0.08	28	1.4	41	0.18
**rcSO_2_ (%)**	79.6	6.3	15	68.7	10	28	-4.4	40	<0.0001*
**SpO_2_ (%)**	99	1.09	15	91	6.01	28	-6.4	30	<0.0001*
**FTOE**	0.19	0.06	15	0.26	0.08	28	-2.5	35	<0.02*
**AVO_2_ (%)**	19.36	6.32	15	22.85	6.89	28	-1.7	31	0.1
**Apgar (1 min)**	8.2	0.63	10	7.7	0.73	27	0.9	35	0.39
**Hgb**				15.9	1.6	28			
**Sex**							χ^2^ = 7.5	1	<0.01*
**Male**			4 (25%)			19 (68%)			
**Female**			12 (75%)			9 (32%)			
**Race/Ethnicity**							χ^2^ = 10.2	3	<0.02*
**Caucasian**			7 (47%)			6 (21%)			
**Latino**			3 (20%)			19 (68%)			
**Asian/Other**			6 (38%)			3 (11%)			

Group comparisons employed either two-sample t-tests or chi-square tests. P-values were 2-sided. Hgb levels were only obtained in the CHD group.

CHD = congenital heart disease; Hgb = hemoglobin; rcSO_2_ = regional cerebral oxygen saturation; SpO_2_ = preductal peripheral oxygen saturation; FTOE = fractional tissue oxygen extraction; AVO_2_ = arterial venous oxygen difference.

*p<0.05.

#### Descriptive statistics

We examined univariate distributions and bivariate correlations for our independent and dependent variables, and plotted bar graphs of cerebral oxygen saturation and peripheral arterial oxygen saturation values across time to aid the identification of outlier values. For these descriptive analyses, we calculated the mean values of cerebral oxygen saturation and peripheral arterial oxygen saturation by averaging all values during the last 2-minutes of supine and first 2-minutes of sitting values for each participant. We used a Pearson’s correlation coefficient to assess the association of cerebral oxygen saturation with peripheral arterial oxygen saturation separately within the CHD and control groups.

#### Hypothesis testing

In the presence of cerebrovascular stability, cerebral oxygen saturation (as indexed by cerebral oxygen saturation values) should vary minimally after a postural change. Therefore, we used the change in cerebral oxygen saturation with posture to assess the integrity of cerebrovascular stability. We hypothesized that CHD infants would have lower cerebral oxygen saturation than control infants and that the association of cerebral oxygen saturation with posture would differ across groups. We used a generalized estimating equation to account for the repeated measures of cerebral oxygen saturation values over time and to test our *a priori* hypotheses. The dependent variable was *cerebral oxygen saturation*, independent variables were *group and posture*, *and covariates were postconceptional age (the sum of gestational age at delivery and postnatal age)*, *postnatal age*, *ethnicity*, *sex*, *and* preductal *peripheral arterial oxygen saturation*. The significance of the main effect of group tested our hypothesis of reduced cerebral oxygen saturation in CHD infants. We then added a group-by-posture interaction to this base model to test our hypothesis of cerebrovascular instability in CHD infants.

We entered covariates stepwise to ensure the stability of the parameter estimates for our effects of interest. We entered preductal peripheral arterial oxygen saturation last into the model to assess whether the hypothesized reduction in cerebral oxygen saturation in the CHD group was disproportionate to the level predicted by peripheral arterial oxygen saturation_._ We also assessed the constancy of parameter estimates for our measures of cerebrovascular instability and cerebral oxygen saturation with and without the preductal peripheral arterial oxygen saturation term in the model.

#### Post hoc analyses

We used t-tests to compare the CHD and control groups on cerebral fractional tissue oxygen extraction (calculated as [preductal peripheral arterial oxygen saturation *minus* cerebral oxygen saturation] *divided by* preductal peripheral arterial oxygen saturation) and arteriovenous oxygen differences (calculated as preductal peripheral arterial oxygen saturation *minus* cerebral oxygen saturation) to understand better the origins of group differences in brain oxygenation and cerebrovascular instability. Admixture of oxygenated and deoxygenated blood at any level will change the resulting cerebral arterial oxygen saturation, but we are controlling for those changes by measuring preductal peripheral arterial oxygen saturation. We also included fractional tissue oxygen extraction separately as a covariate in the generalized estimating equation model that tested our a priori hypotheses to assess whether controlling for fractional tissue oxygen extraction altered cerebral oxygenation or cerebrovascular stability findings (dependent variable was cerebral oxygen saturation; independent variables were group, posture, and group-by-posture interaction; covariates were postconceptional age, ethnicity, sex, preductal peripheral arterial oxygen saturation, and fractional tissue oxygen extraction).

#### Moderator effects

We also examined whether other variables–including postconceptional age, postnatal age, sex, ethnicity, and preductal peripheral arterial oxygen saturation–modified (as a statistical interaction) the effects of posture on cerebral oxygen saturation, or equivalently whether cerebrovascular instability depended on those variables, to ensure that these variables did not influence our cerebrovascular instability findings. Similarly, in a generalized estimating equation model for only the CHD infants, we examined whether having a single ventricle versus biventricular cardiac defect, or the presence of cyanosis (i.e. intracardiac defects causing right to left shunting), modified the association of posture with cerebral oxygen saturation (covariates included postconceptional age, sex, and preductal peripheral arterial oxygen saturation). These models were hierarchically well organized, such that each of the component terms for an interaction was included as main effects.

#### Exploratory analyses

We compared the variances in average cerebral oxygen saturation values between CHD and control infants. We also tested the effects of posture and its interaction with group on peripheral arterial oxygen saturation values to assess whether the cerebrovascular instability effects, as defined when using cerebral oxygen saturation as our dependent measure in the generalized estimating equation analysis, were also present when using preductal *peripheral arterial oxygen saturation* as our dependent measure (independent variables were *group*, *posture*, *and group-by-posture interaction; covariates were postconceptional age*, *ethnicity*, *and sex*). In other words, we assessed whether the postural effects were specific to cerebral oxygen saturation or were a general consequence of systemic hypoxia in the CHD group. Lastly, we used Pearson’s correlation coefficient to assess whether averaged supine and sitting cerebral oxygen saturation values correlated with an index of cerebrovascular instability (the difference between mean supine and mean sitting cerebral oxygen saturation values) to determine whether the degree of cyanosis could account for the degree of cerebrovascular instability. P-values for post hoc and exploratory analyses were reported at a 2-sided significance level of 0.05, unadjusted for multiple comparisons.

#### Missing values

All missing values were excluded from analyses. Preductal peripheral arterial oxygen saturation had 146 missing values and cerebral oxygen saturation had 24 missing values, both out of 2,064 total values.

## Results

### Sample demographics

**Fig 2:**
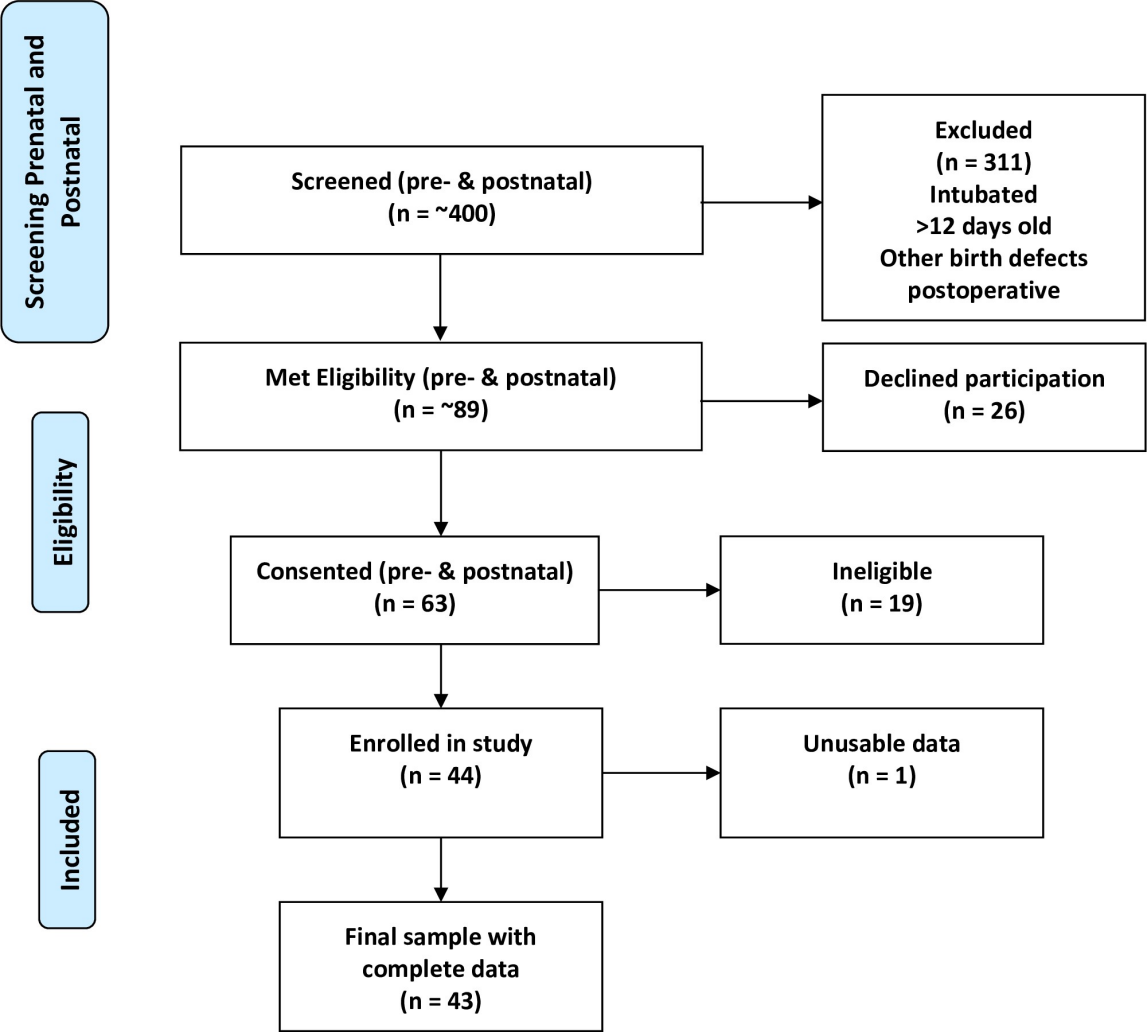
Eligibility flow diagram.

Forty-three infants (28 CHD, 15 healthy controls) completed the study and were included in the statistical analyses. The eligibility flow diagram demonstrates how our final sample was obtained (**Fig 2**). Univariate statistics and group comparisons for infant characteristics are shown in **Table 1**. The two groups matched well on physical and perinatal variables, such as birth weight, length, head circumference, gestational age at birth, and postconceptional age. The groups differed significantly, however, on postnatal age, ethnicity, and sex, with disproportionately more males and younger infants in the CHD group **(Table 1)**. Assessments of CHD infants had to be performed before cardiac surgery, which was soon after birth, yielding a younger group. Parents of control infants were reluctant to return to the hospital for assessments in the first week postpartum, producing an older group. More control infants were born to hospital employees, yielding a higher percentage of Caucasians than in the CHD group, who were more often born to Latino mothers from the community surrounding Children’s Hospital Los Angeles. CHD infants had significantly lower mean cerebral oxygen saturation (68.7% vs. 79.6%, p < .0001) and preductal peripheral arterial oxygen saturation (91% vs. 99%, p < .0001) compared to the controls.

#### CHD defects

Our CHD group consisted of a wide variety of heart defects **(Table 2)**, most being either hypoplastic left heart syndrome (18%) or d-transposition of the great arteries [TGA] (18%), followed by tetralogy of Fallot (7%) and double outlet right ventricle (7%). CHD infants with single compared to biventricular defects were younger (1.5 ± 1.1 vs 3.6 ± 3.4 postnatal days, p<0.03), but did not differ significantly with respect to preductal peripheral arterial oxygen saturation, cerebral oxygen saturation, sex, or postconceptional age.

**Table 2:** Subtypes of cardiac defects.

Subtype (N = 28)	N (%)
**Biventricular Cardiac Defects**	
Aortic Stenosis	1 (3.6%)*
Cor Triatriatum	1 (3.6%)*
D- TGA	5 (17.9%)
DORV	2 (7.1%)
with malposed great arteries (d-TGA type)	
with malposed great arteries (d-TGA type), COA	
Isolated COA	2 (7.1%)*
Shone’s Complex	1 (3.6%)*
Tetralogy of Fallot	2 (7.1%)
Truncus Arteriosus	1 (3.6%)
Ventricular Septal Defect / Interrupted Aortic Arch	2 (7.1%)*
**Single Ventricle Cardiac Defects**	
Double Inlet Left Ventricle	2 (7.1%)
Hypoplastic Left Heart Syndrome	5 (17.9%)
Single Ventricle with Pulmonary Atresia, Heterotaxy	1 (3.6%)
Tricuspid Atresia	3 (10.7%)

COA = Coarctation of the aorta; D-TGA = D-Transposition of the Great Arteries; DORV = Double outlet right ventricle

* = acyanotic cardiac defects.

#### Bivariate correlations

**Fig 3:**
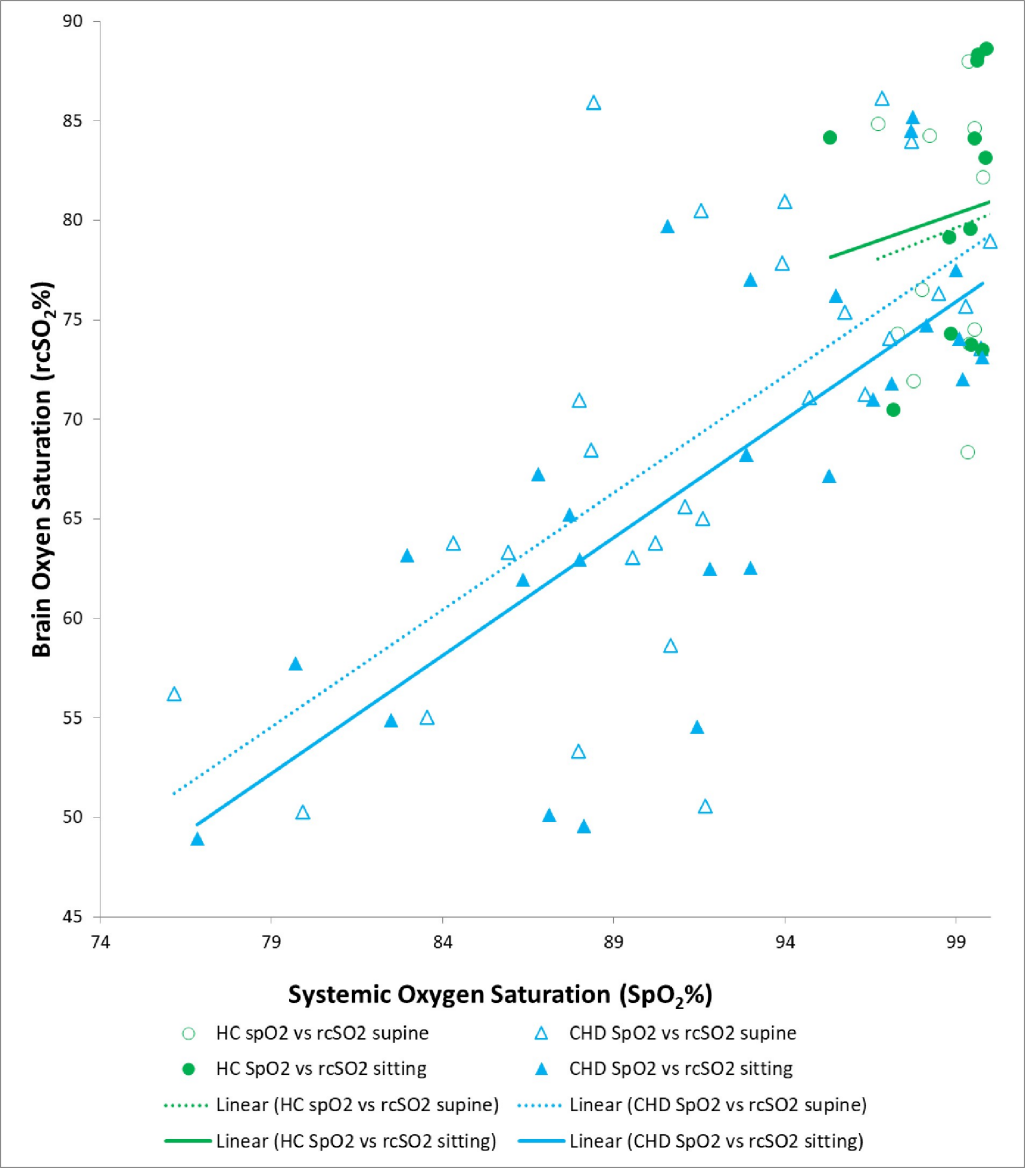
Association of brain oxygen saturation (rcSO_2_) with systemic oxygen saturation for CHD and healthy infants in supine and sitting postures. The significant correlation of rcSO_2_ with SpO_2_ is apparent in each posture within each group. In addition, a difference in mean rcSO_2_ values between the HC and CHD groups is apparent after accounting for the correlation of rcSO_2_ with SpO_2_, in that the regression lines are nearly parallel, though the CHD regression lines extend nearly 10 rcSO_2_ units below the HC regression lines. CHD = congenital heart disease; HC = healthy control; rcSO_2_ = regional cerebral oxygen saturation; SpO_2_ = peripheral oxygen saturation.

**Fig 3** demonstrates the relationship between cerebral oxygen saturation and peripheral arterial oxygen saturation for both subject groups in the supine and sitting posture, as well as their individual linear trend lines. Cerebral oxygen saturation correlated highly with preductal peripheral arterial oxygen saturation (*r* = 0.75, p<0.0001). In an analysis of all infants, the difference in mean cerebral oxygen saturation values across postures correlated significantly with postnatal age (*r* = -0.39, p = 0.01), and postnatal age correlated significantly with group (the CHD infants were significantly younger) **(Table 1)**, indicating that postnatal age is a potential confound in our group comparisons of cerebral oxygen saturation and cerebrovascular instability. We note, however, that the mean cerebral oxygen saturation values across postures did not correlate significantly with postconceptional age (*r* = -0.13, p = 0.43). Arteriovenous oxygen difference and fractional tissue oxygen extraction were independent of sex, gestational age, postnatal age, and postconceptional age. Both arteriovenous oxygen difference and fractional tissue oxygen extraction were more closely associated with cerebral oxygen saturation (r^2^ = 0.69–0.86) than with preductal peripheral arterial oxygen saturation (r^2^ = 0.08–0.22). Fractional tissue oxygen extraction and arteriovenous oxygen difference were collinear with one another (r^2^ = 0.96), so further analyses were performed using only fractional tissue oxygen extraction.

### A priori hypothesis testing

**Fig 4:**
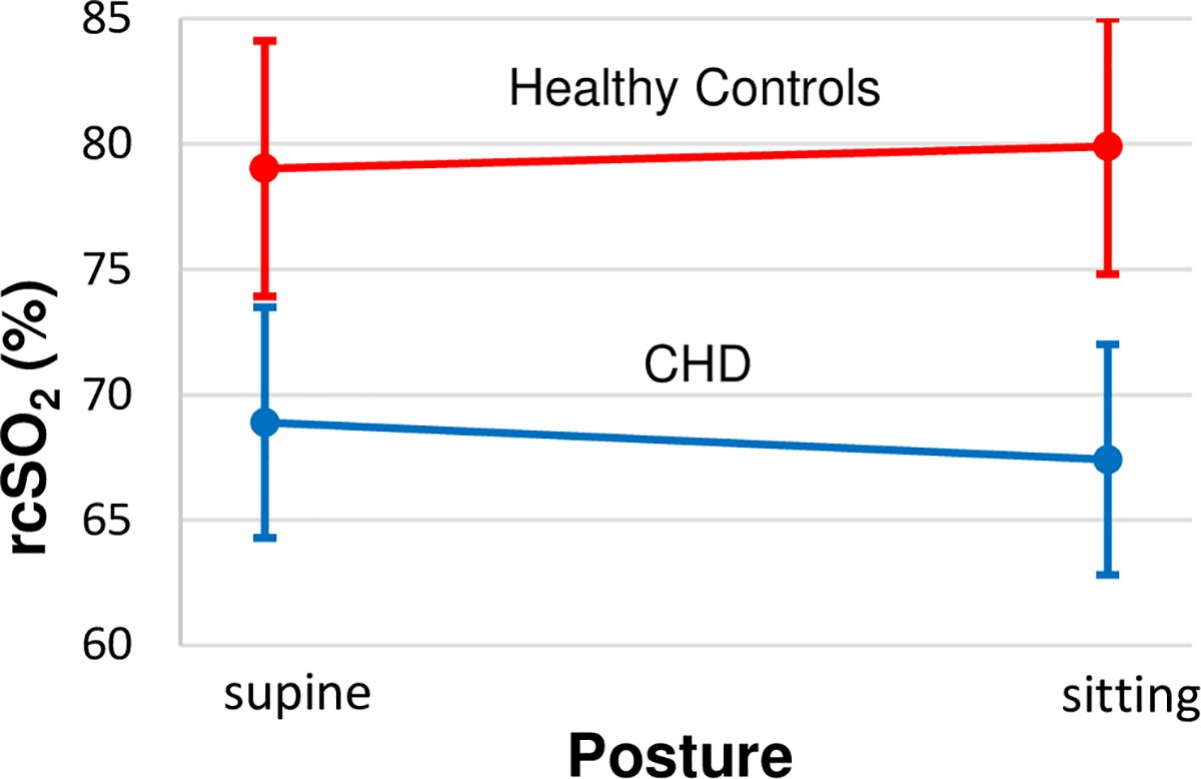
Marginal means for postural effects on cerebral oxygenation. This figure demonstrates the direction of effects for cerebrovascular stability in each group. rcSO_2_ values are least square means estimated from the generalized estimating equation that tested our a priori hypotheses and are adjusted for postconceptional age, sex, ethnicity, and SpO_2_. Brain oxygen saturation decreased from the supine to sitting posture in CHD infants, but it increased slightly in healthy controls. Error bars represent standard error. CHD = congenital heart disease; rcSO_2_ = regional cerebral oxygen saturation.

We detected a significant main effect of group on cerebral oxygen saturation values (β = -9.3; 95% CI = -17.68,-1.00; p = 0.03) **(Table 3)**, indicating that cerebral oxygen saturation was lower in CHD than control infants during both supine and sitting postures (marginal means were approximately 10% lower in the CHD group in both postures: healthy supine 78%, SE = 2.9 vs. CHD supine 69%, SE = 2.1 [Cohen’s *d* = .81]; healthy sitting 80%, SE = 2.7 vs. CHD sitting 68%, SE = 2.2 [Cohen’s *d* = .81]) **(Fig 4)**. This main effect of group on cerebral oxygen saturation values remained significant while covarying for preductal peripheral arterial oxygen saturation values (β = -9.3; 95% CI = -17.68, -1.00; p = 0.03), indicating that the reduction in cerebral oxygen saturation in CHD infants was disproportionate to the levels predicted by their low preductal peripheral oxygen saturation, and when not including preductal peripheral arterial oxygen saturation as a covariate (β = -10.16; 95% CI = -4.11, -0.68; p = 0.02). We note that the magnitude of the group effect was similar across this primary and sensitivity analyses, supporting the regularity and validity of the finding. Nevertheless, given the high correlation of cerebral oxygen saturation with preductal peripheral arterial oxygen saturation, as well as the modest significance level of the group effect on cerebral oxygen saturation, this primary analysis finding should be regarded with caution.

**Table 3:** A priori hypothesis testing.

rcSO_2_	N	β	Standard Error	z	95% CI	p-value
**Postconceptional Age (weeks)**	43	-1.15	0.96	-1.20	(-3.03, 0.73)	0.23
**Age (days)**	43	-0.09	0.62	-0.15	(-1.30, 1.12)	0.88
**SpO**_**2**_ **(%)**	43	0.11	0.06	2.00	(0.002, 0.22)	0.045*
**Posture**						
** Supine**	43	Ref	-	-	-	-
** Sitting**	43	0.88	0.79	1.11	(-0.67, 2.43)	0.27
**Ethnicity**						
** Non-Latino**	21	Ref	-	-	-	-
** Latino**	22	-4.32	3.04	-1.42	(-10.27, 1.63)	0.16
**Sex**						
** Male**	22	Ref	-	-	-	-
** Female**	21	-0.96	3.13	-0.31	(-7.10, 5.18)	0.76
**Group**						
** Healthy control**	15	Ref	-	-	-	-
** CHD**	28	-9.34	4.25	-2.20	(-17.68, -1.00)	0.028*
**Group-by-Posture**		-2.37	0.89	-2.65	(-4.12, -0.61)	0.008*

Generalized estimating equation assessed the associations of group (CHD, healthy control) and posture with rcSO_2_ values. The main effect of group tested our hypothesis of reduced cerebral oxygen saturation, and the group-by-posture interaction tested our hypothesis of cerebrovascular stability in CHD infants. Covariates in the model were postconceptional age, SpO_2,_ sex, and ethnicity.

CHD = congenital heart disease; rcSO_2_ = regional cerebral oxygen saturation; SpO_2_ = preductal peripheral oxygen saturation.

*p < 0.05.

In addition to the main effect, and as hypothesized, we detected a significant group-by-posture interaction (β = -2.4; 95% CI = -4.12, -0.61; p = 0.008), indicating that the *association of posture with cerebral oxygen saturation* (i.e., our operational definition of cerebrovascular instability) varied significantly by group **(Table 3)**. The least squares mean of the interaction revealed that cerebral oxygen saturation did not significantly differ with the change in posture in healthy controls (β = 0.90; 95% CI = -0.80, 2.52; p = 0.27), whereas cerebral oxygen saturation decreased significantly in CHD infants when changing from the supine to sitting posture (β = -1.5; 95% CI = -2.30, -0.68; p = <0.0001) **(Fig 4)**. Parameter estimates for the group-by-posture interaction were similar when excluding preductal peripheral arterial oxygen saturation as a covariate (β = -2.4; 95% CI = -4.11, -0.68; p = 0.006). Including fractional tissue oxygen extraction as an additional covariate did not appreciably alter these findings.

#### Post hoc analyses

Simple T-tests showed that cerebral fractional tissue oxygen extraction was significantly higher in the CHD infants (CHD = 0.26±0.08; healthy = 0.19±.006, T = -2.3, df = 41, p<0.02 [Cohen’s *d* = 0.99]) **(Table 1)**; however, in an analysis of covariance with preductal peripheral arterial oxygen saturation, sex, ethnicity, postconceptional age, and postnatal age as covariates, fractional tissue oxygen extraction was not significantly higher in the CHD group (F = 0.18, p = 0.68). Including fractional tissue oxygen extraction in our statistical model for hypothesis testing revealed that higher fractional tissue oxygen extraction values were significantly associated with lower cerebral oxygen saturation values (β = -106.2; 95% CI = -117.49, -94.87; p = <0.0001), but did not appreciably change our findings for the effect of group or the group-by-posture interaction on cerebral oxygen saturation values (cerebrovascular instability).

#### Moderator effects

We assessed whether covariates moderated cerebrovascular instability by testing the effects of their 2-way interactions with posture on cerebral oxygen saturation values. None were statistically significant, including interactions with postnatal age (β = 0.20; 95%CI = -0.62, 0.97; p = 0.67), postconceptional age (β = 0.10; 95%CI = -0.65, 0.86; p = 0.78), sex (β = -0.70; 95%CI = -2.92, 1.93; p = 0.59), ethnicity (β = 1.1; 95%CI = -0.20, 2.44; p = 0.096), or preductal peripheral arterial oxygen saturation (β = -0.03; 95%CI = -0.17, 0.10; p = 0.58). Therefore, none of these interactions were included in the model testing our *a priori* hypotheses.

Within the CHD group, cerebral oxygen saturation values did not differ according to the type of heart defect (single ventricle vs. biventricular: β = 0.75; 95% CI = -7.39, 8.88; p = 0.86). Type of defect did, however, significantly moderate the effects of posture on cerebral oxygen saturation values (β = -1.5; 95% CI = -2.95, -0.05; p = 0.04) (**Table 4**); least square means indicated that cerebral oxygen saturation dropped significantly in single ventricle, but not biventricular defects when moving from the supine to sitting posture (biventricular supine 68%; 95% CI = 63.1, 73.5; p = <0.0001 vs. biventricular sitting 67%; 95% CI = 62.1, 72.7; p = <0.0001; single ventricle supine 69%, 95% CI = 63.6, 74.5; p = <0.0001vs. single ventricle sitting 67%, 95% CI = 61.4, 71.8; p = <0.0001) when covarying for possible confounds. Infants with cyanotic CHD had significantly lower cerebral oxygen saturation values compared with acyanotic defects (β = -10.78; 95% CI = -16.26, -5.30; p = <0.0001), though cyanosis did not significantly modify the association of posture with cerebral oxygen saturation values (i.e., cyanosis did not moderate cerebrovascular instability) (β = -0.89; 95% CI = -2.65, 0.88; p = 0.33) when adjusting for postconceptional age, systemic oxygenation, sex, and posture (**Table 5**).

**Table 4:** Effects of single ventricle CHD on cerebrovascular stability.

rcSO_2_	N	β	Standard Error	z	95% CI	p-value
**Postconceptional Age (weeks)**	28	-0.23	1.48	-0.16	(-3.13, 2.67)	0.88
**SpO**_**2**_ **(%)**	28	0.08	0.05	1.53	(-0.02, 0.18)	0.13
**Single Ventricle**						
** No**	18	Ref	-		-	-
** Yes**	10	0.75	4.15	0.18	(-7.39, 8.88)	0.86
**Posture**	28					
** Supine**		Ref	-		-	-
** Sitting**		-0.95	0.51	-1.87	(-1.95, 0.05)	0.06
**Sex**	28					
** Male**		Ref	-	-	-	-
** Female**		-0.23	4.57	-0.05	(-9.18, 8.72)	0.96
**Single ventricle-by-posture**	28	-1.50	0.74	-2.03	(-2.95, -0.05)	0.04

Generalized estimating equation assessed the associations of single ventricle vs. biventricular defects and posture with rcSO_2_ values. The main effect of ventricle type tested our hypothesis of reduced cerebral oxygen saturation, and the single ventricle-by-posture interaction tested our hypothesis of impaired cerebrovascular stability in single ventricle CHD. Covariates in the model were postconceptional age, SpO_2,_ and sex.

CHD = congenital heart disease; rcSO_2_ = regional cerebral oxygen saturation; SpO_2_ = preductal peripheral oxygen saturation.

*p < 0.05.

**Table 5:** Effects of cyanotic CHD on cerebrovascular stability.

rcSO_2_	N	β	Standard Error	z	95% CI	p-value
**Postconceptional Age (weeks)**	28	-0.19	1.21	-0.15	(-2.57, 2.19)	0.88
**SpO**_**2**_ **(%)**	28	0.09	0.05	1.61	(-0.02, 0.19)	0.11
**Cyanosis**						
** No**	7	Ref	-		-	-
** Yes**	21	-10.78	2.81	-3.86	(-16.26, -5.3)	<0.0001*
**Posture**	28					
** Supine**		Ref	-		-	-
** Sitting**		-0.84	0.76	-1.11	(-2.34, 0.65)	0.27
**Sex**	28					
** Male**		Ref	-	-	-	-
** Female**		-1.00	3.61	-0.28	(-8.08, 6.09)	0.78
**Cyanosis-by-posture**	28	-0.89	0.90	-0.98	(-2.65, 0.88)	0.33

Generalized estimating equation assessed the associations of cyanotic vs. non-cyanotic defects and posture with rcSO_2_ values. The main effect of cyanosis tested our hypothesis of reduced cerebral oxygen saturation, and the cyanosis-by-posture interaction tested our hypothesis of cerebrovascular instability in cyanotic CHD. Covariates in the model were postconceptional age, SpO_2,_ and sex.

CHD = congenital heart disease; rcSO_2_ = regional cerebral oxygen saturation; SpO_2_ = preductal peripheral oxygen saturation.

*p < 0.05.

#### Exploratory analyses

Replacing cerebral oxygen saturation with preductal peripheral arterial oxygen saturation as the dependent variable in our statistical model yielded nonsignificant effects for both posture (β = 0.24; 95% CI = -0.28,.76; p = 0.36) and the group-by-posture interaction (β = -0.37; 95% CI = -1.28,0.54; p = 0.43) (**Table 6**). Similarly, the CHD group did not exhibit significant effects of posture on preductal peripheral arterial oxygen saturation (β = -0.13; 95% CI = -0.88,0.76; p = 0.74) (**Table 7**). These findings show that the postural change did not affect peripheral arterial oxygen saturation values and, more importantly, that the correlation of posture with preductal peripheral arterial oxygen saturation values did not differ significantly across groups. In other words, group differences in the effects of posture on cerebral oxygen saturation values (cerebrovascular instability effects) were specific to brain oxygenation and did not simply mirror effects that CHD had on oxygen saturation in the periphery. Lastly, average cerebral oxygen saturation did not significantly correlate with the difference in supine and sitting cerebral oxygen saturation values (*r* = -0.22, p = 0.16), indicating that the degree of cerebral hypoxia did not account for the degree of cerebrovascular instability.

**Table 6:** SpO_2_ moderation of cerebrovascular stability effects.

SpO_2_	N	β	Standard Error	z	95% CI	p-value
**Postconceptional Age (weeks)**	43	-0.35	0.63	-0.55	(-1.57, 0.88)	0.58
**Age (days)**	43	-0.22	0.45	-0.50	(-1.10, 0.65)	0.61
**Posture**	43					
** Supine**		Ref	-	-	-	-
** Sitting**	43	0.24	0.26	0.92	(-0.28, 0.76)	0.36
**Ethnicity**						
** Non-Hispanic**	21	Ref	-	-	-	-
** Hispanic**	22	-2.92	1.94	-1.51	(-6.72, 0.88)	0.13
**Sex**						
** Male**	22	Ref	-	-	-	-
** Female**	21	-3.54	1.81	-1.95	(-7.09. 0.01)	0.05
**Group**						
** Health control**	15	Ref	-	-	-	-
** CHD**	28	-8.72	2.36	-3.69	(-13.35, -4.09)	<0.0001*
**Group-by-Posture**		-0.37	0.46	-0.80	(-1.28, 0.54)	0.43

A generalized estimating equation assessed the associations of group (CHD, healthy control) and posture with SpO_2_ values (the dependent variable). Covariates were postconceptional age, sex, and ethnicity. The group-by-posture interaction tested SpO_2_’s influence on cerebrovascular stability.

CHD = congenital heart disease; rcSO_2_ = regional cerebral oxygen saturation; SpO_2_ = preductal peripheral oxygen saturation.

*p < 0.05.

**Table 7:** Effects of posture on SpO_2_ effects in the CHD infants.

SpO_2_	N	β	Standard Error	z	95% CI	p-value
**Postconceptional**	28	-1.42	1.18	-1.20	(-3.73, 0.89)	0.23
** Age (weeks)**						
**Age (days)**		-0.22	0.68	-0.32	(-1.55, 1.12)	0.75
**Posture**	28					
** Supine**		Ref	-	-	-	-
** Sitting**		-0.13	0.38	-0.34	(-0.88, 0.63)	0.74
**Ethnicity**	28					
** Non-Hispanic**		Ref	-	-	-	-
** Hispanic**		-5.97	2.51	-2.38	(-10.89, -1.05)	0.02
**Gender**	28					
** Male**		Ref	-	-	-	-
** Female**		-6.36	2.16	-2.94	(-10.60, -2.12)	0.003

A Generalized Estimating Equation assessed the associations posture with SpO_2_ values (the dependent variable). Covariates were postconceptional age, postnatal age, sex, and ethnicity.

CHD = congenital heart disease; SpO_2_ = preductal peripheral oxygen saturation.

*p < 0.05.

## Discussion

To the best of our knowledge, this is the first study to report evidence of cerebrovascular instability noninvasively in CHD compared with healthy infants. As hypothesized, we detected a significantly altered cerebral oxygen saturation response post-postural change in CHD infants compared to controls. Controlling for group differences in preductal systemic oxygen saturation did not appreciably affect these findings. Although statistical power was limited in assessing differences among heart defects, we found significantly more cerebrovascular instability in single ventricle than in biventricular defects. Cerebral oxygen saturation levels were 70–80% in controls, comparable to previously reported NIRS-based measures in healthy infants [21–23]. Cerebral oxygen saturation values of 50–60% in our CHD infants were much lower than in our healthy infants, though consistent with prior NIRS-based studies of CHD infants [24–27].

### Effects of systemic hypoxemia

Most CHD infants were systemically hypoxemic, with average preductal peripheral arterial oxygen saturation levels of 91% compared to healthy control values of 99%. Posture did not significantly influence preductal peripheral arterial oxygen saturation levels (**Table 4**), indicating that postural effects were specific to brain oxygenation, and demonstrating the independent regulation of central and preductal peripheral oxygen saturation. Preductal peripheral oxygen saturation correlated significantly with NIRS-based cerebral oxygen saturation, however, suggesting that low cerebral oxygen saturations could have derived in part from low peripheral oxygen saturations. While lower preductal peripheral oxygen saturation accounted for most of the lower supine cerebral oxygen saturation in CHD infants, cerebral oxygen saturation remained significantly lower in CHD infants following postural change even when controlling for preductal peripheral arterial oxygen saturation. Moreover, sustained central hypoxia, whether it derives from lower peripheral oxygenation or is independent of it, can potentially damage the brain and impair its development.

### Cerebrovascular instability

Cerebral oxygen saturation values decreased significantly in preoperative CHD infants but increased slightly in the healthy control infants following the postural change, suggesting the presence of cerebrovascular instability in the CHD group. Our finding is similar to other investigators who found significantly lower cerebral oxygen saturation values after a tilt maneuver in preterm infants compared to controls at 5–6 months corrected gestational age [10]. That study, however, compared preterm and term infants at older ages, 2 weeks-6 months corrected gestational age, not at birth. Another report found no significant change in cerebral oxygen saturation in healthy term infants in the supine position, which potentially supports our finding of cerebrovascular stability in healthy infants [28]. Relative to the overall 10% reductions in systemic oxygen saturation levels in our CHD infants compared with controls, their degree of cerebrovascular instability following the postural change was small: cerebral oxygen saturation values declined on average by 2% in CHD infants when changing from a supine to sitting posture (p<0.0001) and increased by 2% in healthy infants following the postural change (p = 0.31). Although we did not find significant effects of cyanosis on cerebrovascular instability, we believe that examining older infants exposed to hypoxemia more chronically may yield different results.

### Possible mechanisms

The cerebral hypoxia and cerebrovascular instability we detected in CHD infants may have related causes. Indeed, we hypothesize that in-utero hypoxemia in CHD infants stimulates physiological changes that can cause cerebrovascular instability. Prior studies in human adults, for example, have shown that hypoxia acutely dilates cerebral vessels [29] to increase cerebral blood flow, then sub-acutely increases Hgb synthesis to improve oxygen-carrying capacity [30], and then chronically stimulates angiogenesis, which in turn increases cerebral blood volume, boosts oxygen content, and reduces red blood cell transit time to improve oxygen delivery and extraction [30]. Increasing evidence suggests that the cerebrovascular responses of CHD infants to cerebral hypoxia may follow a similar compensatory sequence. Perfusion MRI studies, for example, have reported significantly lower cerebral oxygenation [31], diminished placental blood flow [32], reduced oxygenation [32], and increased cerebral blood flow [19, 33] in CHD compared with control fetuses.

Taken together, these prior studies suggest the presence, *in utero*, of chronically reduced perfusion, oxygen delivery, and tissue oxygenation, and a compensatory vasodilation and increased cerebral blood flow, in CHD fetuses. In addition, several magnetic resonance spectroscopy studies of CHD infants have reported increased cerebral lactate, which is a product of hypoxia and is a powerful vasodilator [34–38]. Evidence for a compensatory subacute synthesis of Hgb is more limited, though cross-sectional studies of CHD infants have reported an inverse correlation of erythropoietin levels with arterial oxygen content [39–42], consistent with a compensatory synthesis of Hgb to improve oxygen-carrying capacity. Lastly, postmortem studies of CHD compared with control fetuses reported significantly higher expression of angiogenic factors in the brain [43] and myocardium [44], and studies in cyanotic and single ventricle CHD infants reported higher plasma levels of angiogenic factors compared with healthy infants [45, 46], consistent with the presence of angiogenesis in utero and infancy to compensate for chronic cerebral hypoxia.

Although we did not measure angiogenic factors, we theorize that these putative acute, subacute, and chronic effects of hypoxia may contribute to changes in vascular reactivity and may limit vascular responses during a drop in systemic blood pressure, thereby causing cerebrovascular instability, perhaps even more so in single ventricle CHD. We theorize that chronic, profound cerebral vasodilation in response to hypoxia may directly limit cerebrovascular responses in CHD infants, possibly because the vessels are already maximally dilated, or because vascular tone or reactivity is altered by the chronic dilation. Speaking somewhat against the hypothesis, however, was our finding that baseline cerebral oxygen saturation did not correlate significantly with an index of cerebrovascular instability created for each infant (posture-induced changes in mean cerebral oxygen saturation levels). In addition, arteriovenous oxygen difference values in CHD infants were similar to values in healthy controls, arguing against the presence of a significant compensatory hyperemia (since arteriovenous oxygen difference = [cerebral blood flow X oxygen-carrying capacity] / cerebral metabolic rate). Finally, it is possible that cerebral perfusion pressures in CHD infants (because of their shunted circulation) are at the lower range of values at which cerebrovascular responses holds cerebral blood flow constant; below that range, cerebral blood flow correlates linearly with pressures [47], and the drop in systemic blood pressure with the postural change could have dropped cerebral perfusion pressures, then cerebral blood flow and, by extension, the associated cerebral oxygen saturation values. We did not, however, acquire systemic blood pressure for all CHD and healthy infants to formally test this possibility.

### Potential consequences

Regardless of any causal relationship between cerebral hypoxia and cerebrovascular instability, chronic hypoxia and cerebrovascular instability may render the brains of CHD infants vulnerable to even subtle changes in oxygen saturation and blood flow. The head-up tilt used in this study is a quite innocuous stimulus to which babies will be exposed dozens of times a day; nevertheless, it generated a 9.3% average difference in saturation points even after correction for peripheral arterial oxygen saturation differences. Furthermore, the uncorrected (for preductal peripheral arterial oxygen saturation) cerebral oxygen saturation values are probably more relevant for brain health.

We can assume that the control infants had a healthy response, even if it is immature and the CHD infants differed from that response. Generally, infants are sat up and put down multiple times a day, which is a significant stimulus. We do not know the physiologic cause, but CHD infants are clearly responding differently than the controls in their cerebral oxygenation in response to postural changes. If they cannot regulate their brain saturation properly to normal postural changes, particularly in the context of chronic cerebral hypoxia in any position, they may be vulnerable to brain injury and disordered brain growth. Indeed, increasing evidence suggests that chronic hypoxia may contribute to the high rates of brain injury reported in CHD fetuses and infants, long before surgical correction of their heart defects. These injuries include elevated rates of white matter ischemia, infarct, and hemorrhage [25, 48–52], and delayed anatomical maturation of cortical sulci and gyri [25, 53–54]. Moreover, reports demonstrate the lower cerebral oxygenation significantly correlates with decreased grey matter and gyrification in CHD infants [55] and intraventricular hemorrhage and increased risk of death in preterm infants [56, 57].

### Cerebral tissue extraction

Unadjusted fractional tissue oxygen extraction values were significantly higher in CHD compared with control infants (p<0.02), indicating that CHD newborns either have increased cerebral metabolic rate or decreased oxygen delivery. Prior studies have reported higher fractional tissue oxygen extraction in term preoperative CHD infants compared with controls, who had fractional tissue oxygen extraction values comparable to our infants [27, 58–60]. We also found that higher fractional tissue oxygen extraction values were strongly associated with lower cerebral oxygen saturation levels when accounting for the effects of group and preductal peripheral arterial oxygen saturation on cerebral oxygen saturation values (p = <0.0001), consistent with findings from a study of CHD infants that fractional tissue oxygen extraction increased with declining venous oxygen content [61], a measure akin to NIRS-based cerebral oxygen saturation values.

### Limitations

Our method for assessing cerebrovascular instability made several assumptions that remain unproven. First, rcSO_2_ is a measure of cerebral oxygen saturation, not cerebral blood flow. Its use in our study and other studies to estimate cerebral blood flow assumes that cerebral metabolic rate and oxygen extraction were similar across groups and across postures in each infant, thereby ensuring that NIRS measures would be proportional to cerebral blood flow. We believe that cerebral metabolic rate and oxygen extraction were similar across groups because the infants were calm during the postural change. The assumption that NIRS-based regional oxygen saturation approximates cerebral venous saturation has inherent limitations related to inter-subject differences in venous and arterial blood volume. However, there is no a priori reason to suspect systematic differences in tissue blood volumes in patients with single ventricles or any other CHD [62]. We note that prior studies have validated NIRS-based measures against independent measures of cerebral blood flow [12, 63–65], providing general support for the assumptions underlying the use of NIRS as a surrogate for cerebral blood flow. Our use of postural changes to assess cerebrovascular instability also assumed that the change in posture from supine to sitting produced a reliable change in arterial blood pressure that was similar across CHD and control infants. Postural changes have produced reliable blood pressure changes in older children and adults [66–68] and will require future studies for validation in infants. Our method also assumed that posture-induced changes in cerebral oxygen saturation measured by NIRS were induced by fluctuations in blood pressure, and not by other potential differences between CHD and healthy infants, such as group differences in venous engorgement, blood carbon dioxide levels, changes in intrathoracic or abdominal pressure, level of alertness (and associated alterations in cerebral metabolic rate), shunts through abnormal intra- and extracardiac connections, atrial filling pressures, and blood volume distribution. Future studies would benefit from accurate measures of cardiac output to address some of the limits. Regardless of the ultimate sources of signal in this measure, however, these differences across CHD and control infants are interesting and warrant further investigation.

Other limitations of our study include the heterogeneity of the heart defects and confounders, such as significant differences between CHD and healthy control groups on ethnicity and postnatal age. However, including these demographic variables as covariates did not appreciably alter our findings. Although our institution performs a high volume of CHD surgeries, neonates with CHD are extremely difficult to recruit, for many reasons: parents are stressed from their newborn’s diagnosis and impending surgery; the infants are often too ill for our study procedures, or they are intubated and unable to undergo the procedures. Moreover, stratifying by CHD cyanosis or not would still include heterogeneous structural defects within the cyanotic and acyanotic groups. Stratifying on the basis of structural defect would require extremely large numbers that would not be feasible without a large, multi-center study. Thus, as this was an initial study of cerebrovascular instability in CHD infants, we had little choice but to recruit patients with a mix of CHD types in order to reach our required sample size. Lastly, our study’s generalizability is limited, since most participants were from the Latino CHD population in the Los Angeles metropolitan area.

## Conclusions

Taken together, our data show that cerebral oxygenation declines with a postural change in CHD infants, thereby providing evidence for the presence of cerebrovascular instability. Particularly in the context of chronic cerebral hypoxia, this instability may render the brains of CHD infants at risk for brain injury or disordered development. Our noninvasive method for assessing cerebrovascular instability permitted the evaluation of both CHD and healthy infants, and it succeeded in capturing predicted group differences of cerebral NIRS measures with changes in infant posture. This technique makes possible future longitudinal studies of CHD and healthy infants in order to assess the associations of cerebral hypoxia and cerebrovascular instability with long-term neurodevelopmental and brain imaging outcomes. Identifying the physiologic vulnerabilities and mechanisms that cause cerebrovascular instability may help tailor care and interventions that reduce associated brain injuries and improve long-term neurodevelopmental outcomes in these vulnerable infants.

## Supporting information

S1 Data (XLSX)
